# A New Tool for Investigating the Functional Testing of the VOR

**DOI:** 10.3389/fneur.2013.00165

**Published:** 2013-10-25

**Authors:** Paolo Colagiorgio, Silvia Colnaghi, Maurizio Versino, Stefano Ramat

**Affiliations:** ^1^Bioengineering Laboratory, Department of Computer, Electrical and Biomedical Engineering, Università degli Studi di Pavia, Pavia, Italy; ^2^Neurorehabilitation Unit, Fondazione Istituto Neurologico Nazionale C. Mondino, Pavia, Italy; ^3^Brain and Behavioral Sciences, Università degli Studi di Pavia, Pavia, Italy

**Keywords:** functional vestibular testing, head impulse test, dynamic visual acuity, compensatory saccades, semicircular canals function

## Abstract

Peripheral vestibular function may be tested quantitatively, by measuring the gain of the angular vestibulo-ocular reflex (aVOR), or functionally, by assessing how well the aVOR performs with respect to its goal of stabilizing gaze in space and thus allow to acquire visual information during the head movement. In recent years, several groups have developed clinical and quantitative approaches to functional testing of the vestibular system based on the ability to identify an optotype briefly displayed on screen during head rotations. Although the proposed techniques differ in terms of the parameters controlling the testing paradigm, no study has thus far dealt with understanding the role of such choices in determining the effectiveness and reliability of the testing approach. Moreover, recent work has shown that peripheral vestibular patients may produce corrective saccades during the head movement (covert saccades), yet the role of these eye movements toward reading ability during head rotations is not yet understood. Finally, no study has thus far dealt with measuring the true performance of their experimental setups, which is nonetheless likely to be crucial information for understanding the effectiveness of functional testing approaches. Thus we propose a new software and hardware research tool allowing the combined measurement of eye and head movements, together with the timing of the optotype on screen, during functional testing of the vestibulo-ocular reflex (VOR) based on the Head Impulse Test. The goal of such tool is therefore that of allowing functional testing of the VOR while collecting the experimental data necessary to understand, for instance, (a) the effectiveness of the covert saccades strategy toward image stabilization, (b) which experimental parameters are crucial for optimizing the diagnostic power of the functional testing approach, and (c) which conditions lead to a successful reading or an error trial.

## Introduction

The vestibular system provides our brain with information on the movement of the head using the semicircular canals and the otoliths to transduce head angular velocity and linear acceleration, respectively. Such information is used by the brain to maintain balance and control posture, to program corrective responses, regulate other body rhythms, and to drive the movement of the eyes in order to stabilize vision during head movements through the vestibulo-ocular reflex (VOR).

During head rotations, in order to maintain gaze stable on a stationary target, the healthy angular vestibulo-ocular reflex (aVOR) rotates the eyes with the same velocity and amplitude as the head, but in the opposite direction. Based on these considerations the aVOR may be quantitatively evaluated in the laboratory using specialized equipment for recording the movement of the eyes and of the head and thus computing its gain (eye velocity/head velocity), which equals one in healthy subjects (1). Obviously, eye movement recording is a relatively complicated laboratory procedure requiring specific technical knowledge and relatively expensive equipment, both aspects that limit the use of such approach to only a few laboratories.

Functional testing of the aVOR is an alternative diagnostic tool for identifying peripheral vestibular deficits while avoiding the need to measure the movement of the eyes.

The basic idea behind the current functional testing approach is extremely simple: the function of the VOR is that of stabilizing the image of the seen world on the retina during head movements in order to allow clear vision. Its failure to appropriately do so causes retinal slip, and even a few degrees per second of image movement on the retina seriously deteriorate vision. Thus, several years ago Miller et al. (2) proposed to evaluate the VOR while viewing a stationary optotype during head rotations as a tool for diagnosing vestibular deficits. Several clinical approaches were developed in the eighties and nineties based on high frequency active oscillation of the head (3–6). Mostly these approaches were considered as having limited validity as they were unable to deal with the contribution to reading that may be provided by pursuit eye movements at the slowing of the head during sinusoidal motion. The development of computerized systems in which the letter could be displayed as a function of head velocity (7–9) determined a significant step forward toward defining a reliable test of the functionality of the VOR whilst not recording eye movements.

In the study by Herdman and colleagues (8), subjects had to report the orientation of a letter E optotype that was briefly displayed on a computer screen when head velocity during active head rotations in the horizontal plane was between 120 and 180°/s. The subject’s best visual acuity (Dynamic Visual Acuity, DVA) is sought by decrementing the optotype size by 0.1 logMAR each time and counting misreported orientations out of the five impulses imposed for each size. The effectiveness of the test using active head movements though, is limited since, after the acute phase of a vestibular damage, patients may have learned to use the available information, i.e., the efference copy of the commands to neck muscles, to activate predictive mechanisms driving compensatory eye movements (10, 11). Moreover, chronic patients develop compensatory mechanisms such as adaptation (based on the residual vestibular input) and/or substitution (based on visual and proprioceptive sensory inputs), which are especially effective with head movements having a low frequency content.

Significant progress then was determined by the use of passive head movements (12, 13), based on the rationale of the head impulse test (HIT) clinical technique (14). With such test brief, high angular acceleration (2000–6000°/s^2^) head rotations drive the contralesional semicircular canal afferent firing into inhibitory cutoff. The lack of further afferent firing modulation ability in the inhibitory direction from the healthy side is revealed clinically by the production of one or more overt saccades (i.e., occurring at the end of head rotation) to recover fixation (see Figure 5). When head impulses are imposed during DVA testing the inhibitory cutoff reveals the under-compensatory angular VOR, thus causing retinal slip and preventing clear vision of the optotype, even in chronic patients.

Recent work has been devoted to improving the DVA approach by selecting head velocities above 150°/s, using Landolt rings in eight orientations to reduce the probability of guessing the correct answer and shortening the visual acuity threshold finding procedure using an adaptive algorithm (15).

Other approaches have recently been proposed, such as the Gaze Stabilization Test (GST), which evaluates the aVOR by measuring the maximum head velocity that a subject is able to achieve while still being able to correctly identify the orientation of a fixed-size optotype letter “E” during active head movements (16, 17). Our group has recently developed and tested (18, 19) a functional head impulse testing device (HITD) to assess vestibular function based on the ability of a subject to read a static visual acuity (SVA)-normalized optotype flashed on the screen when head angular acceleration overcomes thresholds ranging 2000–6000°/s^2^.

One reason for the present work stems from the observation that several relevant parameters of the functional testing approach, such as the best timing of the visual display, of its duration on screen or its temporal relationship to the head movement, significantly differ both with the different described techniques (i.e., DVAT, GST, and HITD) and within the DVAT approach itself when carried out by the different cited groups or studies. Certainly a true understanding of these parameters and their optimization for diagnostic purposes would be important for establishing a reliable and easy to use diagnostic approach.

On the other hand, recent work by Weber and colleagues (18, 19) investigating the video head impulse test [vHIT, Ref. (20)] aimed at measuring non-invasively the gain of the VOR during HIT, found that vestibular patients could produce compensatory saccades during the head movement, which they called “covert” saccades (see Figure 5), that would likely be missed by the clinician during clinical HIT. Although such compensatory “covert” saccades have been described in the literature (21–23), their functional consequences, i.e., whether they are effective at improving gaze stabilization during the head movement, and thus the ability to correctly identify the stimuli in functional testing paradigms, or whether their function is that of bringing the eyes back on the fixation point sooner than with the delayed “overt” saccade yet without improving vision while in motion, has never been investigated so far.

Furthermore, when working with commercial operating systems (OS) the timing variability related to the screen refreshing rate, the acquisition and processing software performance, CPU load, and the known other factors related to a non-real-time OS, introduce uncertainty about the adherence of the testing procedure to the intended protocol.

Thus, the goal of the present study was that of developing a new research tool allowing the synchronized recording of eye and head movements, together with a feedback on visual display timing, which we believe are crucial information to truly understand functional aVOR testing, to study the effectiveness of covert saccades toward image stabilization and to define which experimental parameters are critical for optimizing the diagnostic power of functional aVOR testing.

## Materials and Methods

### Overview

The project moved from the research needs above described and thus aimed at integrating our HITD (24, 25) with the EyeSeeCam video-oculography system (26) available in our lab. The choice of such eye movement recording tool was driven by the need for a lightweight device, thereby limiting the consequences of inertial forces, while avoiding the scleral search coil for the inevitable disruption of visual clarity it implies. The experimental paradigms were then developed as Matlab (MATLAB R2009b, The MathWorks, Inc.) scripts handling both the stimulation and data acquisition through data structures made available online by the EyeSeeCam software package in the Matlab workspace.

#### Head impulse testing device

The HITD exploits a head mounted MEMS gyroscope to measure head angular velocities and differentiates such signal online to compute angular accelerations. The HITD testing procedure (24) is as follows: a patient sits in front of the screen wearing a head mounted sensor. First the SVA is assessed using viewing distance-scaled optotypes displayed on screen. The optotype size used for dynamic testing was then obtained by increasing the SVA size by 0.8 logMAR. Then, while the experimenter standing behind the seated subject manually imposes head impulses, the patient is asked to recognize the optotype that is displayed on screen as the imposed head angular acceleration exceeds a user-defined threshold.

The developed tool may be used for studying any of the described functional VOR testing paradigms (i.e., DVAT, GST, and HITD) and their different implementations described in the literature, yet in the following we will describe the new system implementation of the HITD proposed by our group (24, 25). Thus the goal is to test vestibular function at different head angular accelerations, while facing equally challenging visual stimuli and yet directly measuring eye movements and the timing of visual stimuli on screen, in this specific setting.

#### EyeSeeCam system

The EyeSeeCam system is a modular device allowing to measure eye and head movements thanks to one (or two) head mounted sensors, each consisting of:

– an infrared video camera with two integrated light emitting diodes (IRLEDS) with wavelengths of above 850 nm;
– a six degrees of freedom (6DOF) inertial measurement unit (IMU).

The system is provided with an acquisition software package for the measurement of eye movements by means of an automated online analysis of video streams. The results of these image processing steps are stored into Matlab data (.mat) files for subsequent analysis.

The EyeSeeCam system provides an interface to Matlab that enables three distinct tasks:

– present visual stimuli;
– acquire data made available in the Matlab workspace, based on a time-controlled flow;
– analyze and show recorded data.

The EyeSeeCam is delivered with a standard set of stimulation and analysis Matlab scripts that can be customized to the needs of individual laboratories. Similarly, it is possible to extend the provided set of functions with user-written scripts. The scripts presenting visual stimuli exploit the PsychToolbox-3 (27, 28), which also allows openGL commands. The online communication with the acquisition software is granted by Matlab mex files provided with the system, which allow to access the data and to control the stimuli and experimental paradigms. Up to eight additional analog channels may be acquired and synced with the rest of the data through National Instruments USB data acquisition devices.

The .mat file saved at the end of the acquisition contains synchronized data samples relative to the head movement (provided by the IMU), eye position (video-oculography), and the analog channel representing the photodiode output, stored in a single matrix together with its relative timestamps.

The software workflow and the interaction between the scripts are managed through a graphical user interface (GUI).

The camera frame rate determines the acquisition frequency of the whole system and can be defined by the experimenter, yet it is inversely related to the spatial resolution of each acquired frame, and thus of the resulting angular eye position. For our experiments we have chosen a sampling rate of 220 Hz, which determined frames of 188 × 120 pixels. Our system is monocular and was set to measure movements of the left eye.

### Integration of the HITD and EyeSeeCam systems: VideoHITD

#### Visual stimulus size

For the new HITD test we chose the Landolt C optotype, a ring with a gap measuring 1/5 of the diameter and can be presented in eight possible orientations at 45° increments, for two main reasons. First because its use grants that all visual stimuli are equally challenging while maintaining the probability of a correct answer by chance to 0.125 [it was 0.1 in the original version of the test related to the use of the Sloan letter set Ref. (25)]. Then because such optotype is relatively simple to handle using openGL functions, a software development environment constraint. Furthermore, the use of such approach simplifies the recording of the patient responses, since the answers may be provided easily by coding the orientation of the ring with the keys of a numerical keyboard.

Optotypes are displayed in black over a white background on the monitor in front of the subject (here at 1 m distance) that discerns the orientation and returns the answer using a numerical keyboard modified with arrows pointing in the direction of the gap. The size of the optotype is scaled depending on the distance from the screen based on (1):

(1) $$\mathrm{Size =}\frac{\mathrm{Distance}}{\frac{1}{\mathrm{5*tan}\left(\frac{1}{\mathrm{60}}\right)}\mathrm{*VisualAcuity}}$$

To improve presentation timing, the Landolt ring is pre-built at the maximum size allowed by the screen size with the gap at 0° and stored. Before being presented on screen it is rotated to one of the eight possible orientations following a pseudo-random sequence and scaled with Eq. 1.

Visual acuity in static conditions is tested at the beginning of the experiment by requiring the patient to identify the orientation of a sequence of Landolt C rings. The size of the ring (and its gap) is reduced depending on the subject rate of errors using the QUEST (29) adaptive algorithm, which is also implemented in the Psychtoolbox. The algorithm starts from a value of 1 logMAR and estimates the viewer’s SVA threshold in 20 trials.

#### Visual stimulus and timing

**Figure 1:**
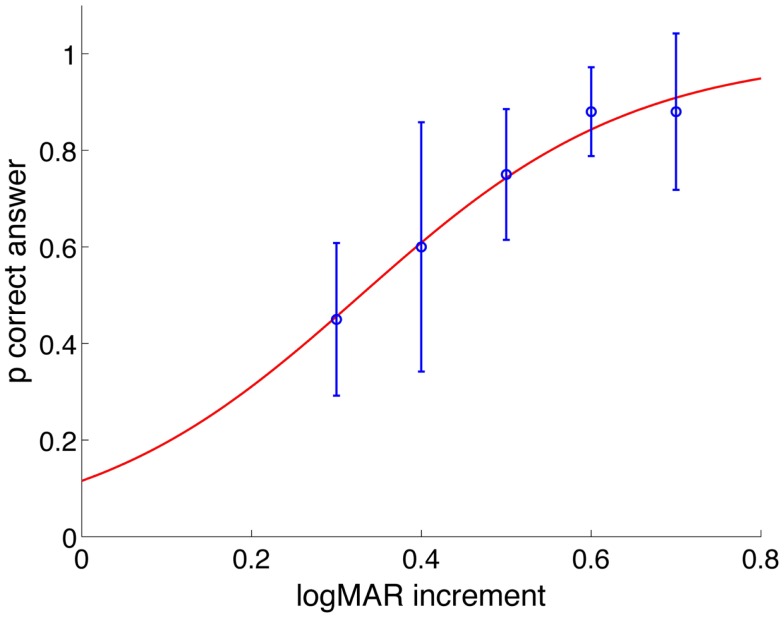
**Probability of correct answers as a function of logMAR increment of all subjects**. Values are fitted with a psychometric logistic function. For increment of 0.5 logMAR and less, the probability of providing a correct answer decreases statistically significantly.

For the HITD test, the size of the stimulus is obtained by increasing the SVA by 0.6 logMAR. Such value is lower than previously used (25) and was chosen based on the assessed reading ability of 10 healthy subjects (visual acuities ranging 0.1–0.7 logMAR) during passive head impulses within a restricted range of accelerations (between 2000 and 4000°/s^2^) and using optotypes with different size increments with respect to their SVA. Ten stimuli were presented for each increment, in a pseudo-random order, and we obtained a psychometric function of the percentage of correct answers as a function of the increments (Figure 1). The increment of 0.5 was the first one whereby the number of errors was significantly greater than zero and we therefore chose to use a 0.6 logMAR increment.

The chosen optotype size will then remain constant through the entire dynamic testing phase. The patient has then to recognize the optotype orientation flashed on screen when the experimenter-imposed head angular acceleration exceeds a selectable threshold. The delay between the overcoming of the threshold and the optotype presentation, as well as its duration on screen may be selected by the experimenter, among multiples of frame duration, computed as the inverse of the screen’s vertical refresh rate.

The user-defined timing of the optotype is obviously only nominal, as it depends on the graphic performance of the computer running the experiment, on the refresh rate of the screen, on the OS scheduling of the running processes. In order to verify the timing of the system, which is a crucial performance for the accuracy of the test, we used a photodiode attached to the test screen for detecting a square that pops up together with the letter in the lower right-hand corner of the screen. The appearance of the square is not perceived by the subject as the entire area is covered by the photodiode assembly box. The output of the photodiode is also captured via the National Instruments USB device connected to EyeSeeCam. The photodiode is a generic RS-Components diode, BPW21, RS part no. 303–719. It has a typical rise-time of 1 μs, which was therefore neglected in the following analyses.

The photodiode data channel, synced with head and eye movement data, thus provides the system with the feedback on the exact timing of the optotype as its output will be high for the whole permanence of the stimulus on screen: a key information for further understanding functional VOR performance in healthy subjects, in patients, and during rehabilitation.

Data from the EyeSeeCam IMU sensor are thus used to measure head angular velocity $\left(\dot{\theta }\right)$ about the vertical axis (see Data analysis) and thus to compute head angular acceleration as:

(2) $$\frac{d{\dot{\theta }}_{z}}{dt}=\frac{{\dot{\theta }}_{z}\left(t\right)-{\dot{\theta }}_{z}\left(t-1\right)}{t_{c}}$$

Then, when head angular velocity overcomes a threshold (e.g., 10°/s) if the acceleration overcomes another threshold (e.g., 300°/s^2^) a thrust is recognized and the optotype is shown.

As previously described the HITD test assesses the functionality of the VOR at different head accelerations so that the imposed head thrusts are classified in acceleration bins (width of 1000°/s^2^ with upper bounds ranging 2000–7000 /s^2^) based on their direction, while an online feedback is provided through the GUI reporting the number of thrusts performed per bin and the corresponding percentage of reading errors, thus helping the experimenter at delivering head thrusts in the whole field of the required accelerations. We chose to impose at least five thrusts per bin and increase such number to eight if the subject performed at least one reading mistake, to improve resolution. Plots of angular velocity and eye velocity relative to the performed thrust are also presented on the feedback GUI.

Clearly, covering the full range of accelerations is a demanding task for both the subject and the experimenter, since at least 60 head impulses are necessary. Yet, although in the context of this research study we have attempted at achieving such task, our previous studies (25) suggest that the range of head accelerations relevant to clinical HITD testing may be reduced to the 3000–6000°/s^2^ bins, which typically requires between 40 and 50 head impulses per subject.

#### Data analysis

During offline processing of the acquired data, eye position is computed using rotation vectors (30). In order to better quantify the stimulus to the horizontal canals which, with proper subject positioning (30° nose down), should lie in the horizontal plane, we chose to consider only the head rotation component lying in that same plane $\left({\dot{\theta }}_{z}\right)$. We thus use the linear acceleration signals from the IMU to assess the sensor’s orientation with respect to gravity and then rotate the angular velocity components from the gyro to compute the angular velocity around the vertical axis. This component is compared with ω*_z_* (horizontal eye velocity).

Velocity gain of the VOR is then calculated as the ratio of the mean eye velocity over the mean head velocity computed over the time interval between head peak acceleration and peak velocity (duration 40 ± 8 ms). The head acceleration value associated with each impulse is computed as the slope of the line that fits five samples of head angular velocity centered at the time of peak acceleration.

A statistical approach to the classification of the results of each subject was previously proposed (25) based on the rate of correct answers and a population of controls.

In the following report of representative experimental results data will be presented, when applicable, as mean ± SD.

The full set of Matlab scripts and functions needed to run the functional VOR testing experimental paradigms based on the EyeSeeCam system and the analyses of the acquired data presented throughout this work are available upon email request to the corresponding author.

## Results

In summary, the goal of such effort was to propose a research tool to allow functional testing of the VOR while collecting the experimental data necessary to face the problem of understanding (a) the effectiveness of the covert saccades strategy toward image stabilization, (b) which experimental parameters are crucial for optimizing the diagnostic power of the functional testing approach, (c) the true timing performances of the system for investigating the causes of a correct vs. a mistaken subject response.

Here we present the results obtained by using such tools in a few possible example scenarios based on the HITD approach: a functional test assessing the ability of the subject to read an optotype briefly displayed on a computer monitor during passive and unpredictable head rotations at different angular accelerations (25).

### System performance

**Figure 2:**
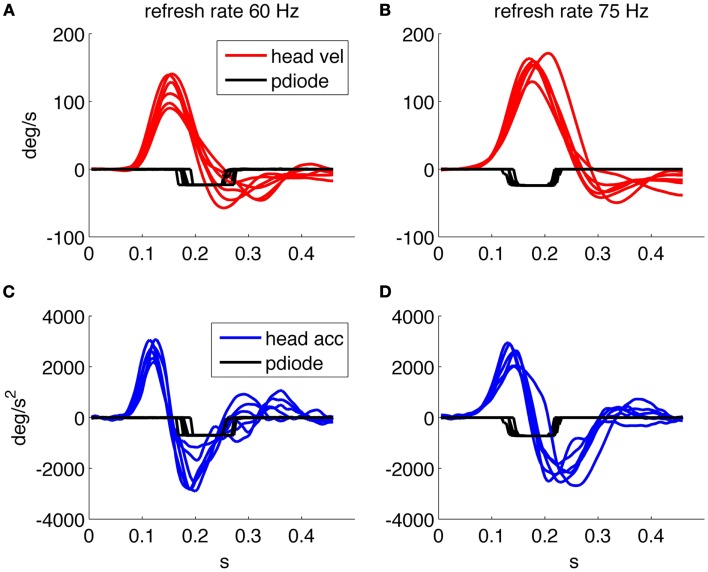
**Timing performance of the system using a screen running at 60 Hz**. **(A)** Head velocity/sensor output. **(C)** Head acceleration/sensor output and one running at 75 Hz. **(B)** Head velocity/sensor output. **(D)** Head acceleration/sensor output. The sensor trace is recorded using a screen mounted photodiode that transduces the presence/absence of the optotype on screen.

Figure 2 shows the timing performance of the system as tested using two different screens: one with refresh rate of 75 Hz and resolution 1280 × 1024 and one with refresh rate of 60 Hz and resolution 1920 × 1080. The thresholds were set to 10°/s for angular velocity and 300°/s^2^ for angular acceleration. The delay of the optotype with threshold detection was set to zero and its on screen duration to 80 or 83 ms, corresponding to six frames at 75 Hz and five frames at 60 Hz vertical refresh rates, respectively.

We then verified the performance using a 75 Hz screen and found a mean optotype delay of 25 ms (25 ± 8 ms) due to both the system performance and the one frame uncertainty related to the asynchronous presentation command. The presentation time is of about 80 ms (80 ± 8 ms) as planned by the experimental paradigm. With the 60 Hz screen the delay was 62 ± 20 ms and the duration on screen was 84 ± 10 ms, thus presenting an unexpectedly longer delay in the presentation of the optotype.

Figure 2A shows that when the optotype is presented with the 60 Hz screen, it can still be displayed while the head velocity crosses 0°/s and changes sign, this can in theory distort the results of the test since even a few millisecond of stable retinal image can allow a correct optotype recognition.

**Figure 3:**
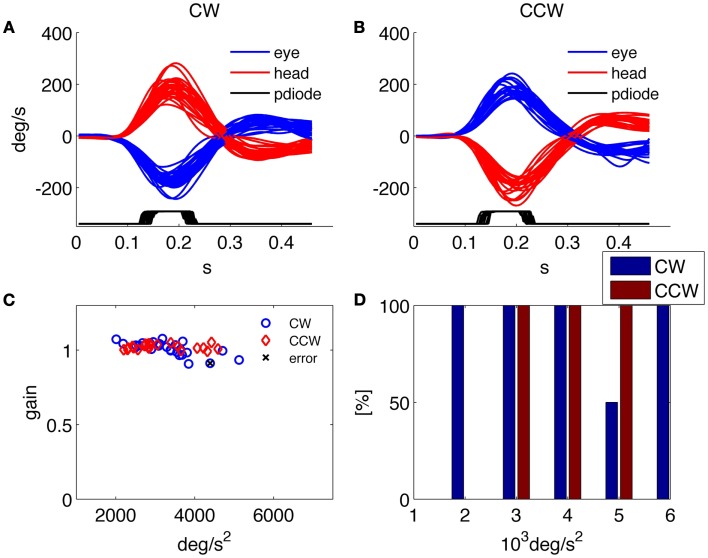
**Results of the HITD test on a healthy subject**. **(A,B)** Head and eye movement for CW and CCW rotations, respectively. **(C)** Plot of VOR gain values computed for each movement. **(D)** Percentage of correct answers for each acceleration bin.

Figures 3A,B shows data relative to an acquisition with the 75 Hz screen of about 60 head thrusts on a healthy subject; the variability of the timing results from the asynchronous command and the discrete timing of the screen quantized by its frame rate.

### Assessment of peripheral vestibular function

**Figure 4:**
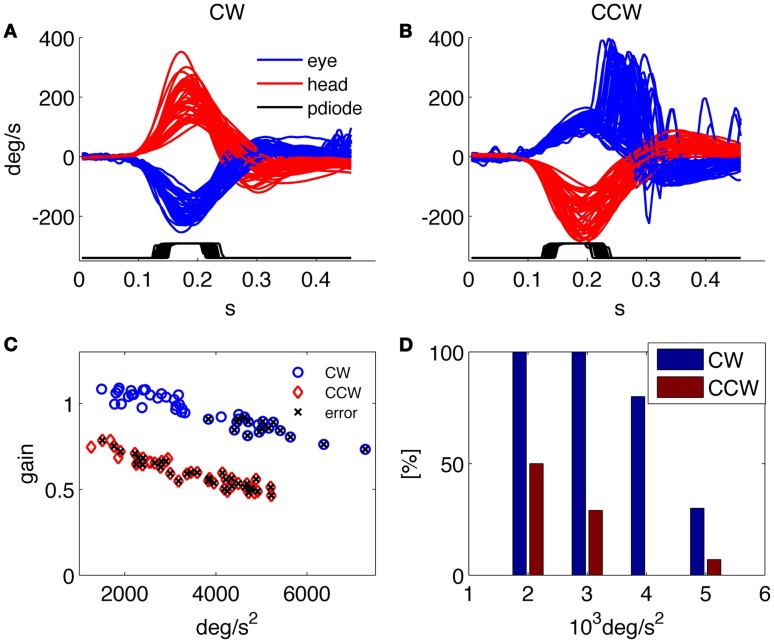
**Results of the HITD test on a patient with vestibular neuritis (left side deficit)**. **(A,B)** Head and eye movement for CW and CCW rotations, respectively. **(C)** Plot of VOR gain values computed for each movement, x indicates wrong reading. **(D)** Percentage of correct answers for each acceleration bin. Overall percentage of correct answers: 18% toward the affected side, 62% toward the contralateral.

Besides providing all the collected data to the examiner in order to allow further analysis and the investigation of specific aspects through customized programs, a predefined set of results is presented by the system at the end of each examination. An example of such standard results panel is shown in Figure 3 for a representative healthy subject, and in Figure 4 for a patient affected by a left side vestibular deficit.

The results report the traces of all recorded head and eye movements for each direction of head rotation [clockwise (CW) and counterclockwise (CCW)] together with the trace showing the timing of the optotype presentation (recorded from the photodiode); the gain of the VOR in response to each head impulse is plotted against its peak head acceleration; and the percentage of correct answers in every direction for each acceleration bin.

The normal subject in Figure 3 was able to read nearly 100% of the presented optotypes and his gain was close to one for each rotation direction and for all tested head accelerations. The low rate of correct answers shown for the 5000°/s^2^ bin in Figure 3D is explained by the results in Figure 3C, showing that only two thrusts were performed for that acceleration bin, one of which caused the single subject’s error throughout the test.

The patient in Figure 4 was recorded 4 days after the onset of a left vestibular neuritis. At rotatory testing (step velocity, acceleration 100°/s^2^ to reach 140°/s velocity; normal gain value: >0.27) he showed a gain value of 0.22 ipsilesionally, and of 0.5 contralesionally. His subjective visual vertical was −4.9°(normal interval: ±2.6°). He had severe problems in identifying the optotype when the head was rotated toward the affected (left) side and a low rate of correct answers was also found during high acceleration rotations toward the contralateral side; indeed, as recently reported (18), the gain decreases with increasing accelerations in both the ipsilesional and the contralesional directions.

### Compensation and recovery strategy

It is now clear that saccades can play an important role in compensating vestibular insufficiencies both in the healthy translational VOR (31, 32) and in the deficient or recovering angular VOR (23) for returning to normal lifestyle (33). One goal for the developed system is to allow studying how the residual VOR and covert saccades are combined in the gaze stabilization task aimed at reading the optotype displayed during head rotation.

**Figure 5:**
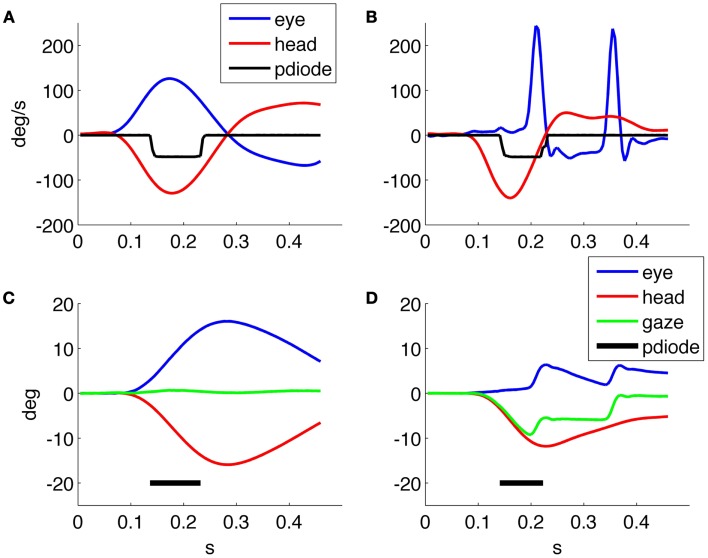
**(A,B)** Head and eye velocity response, and optotype presentation from a normal subject and a patient, respectively, with (left) unilateral vestibular neuritis. **(C,D)** Head, eye, and gaze position, together with optotype timing for the responses in **(A,B)**, respectively.

This may be performed by studying the response to individual head thrusts based on the behavior of the subject’s gaze trace, computed as the sum of the head displacement and eye displacement, during the appearance of the optoptype on screen (Figure 5). Gaze is zero at the beginning of each trial, when the subject is fixating the reference position on screen and with a perfect VOR it will remain still during the thrust. The derivative of gaze is then retinal slip velocity, whose analysis will allow understanding which range of values is compatible with clear vision and when a functional deficit arises.

Figure 5 shows the detail of two representative responses to head impulses triggering the optotype presentation: one from a healthy subject and one from a patient recorded 3 days after the onset of a right vestibular neuritis, when at rotatory testing she showed an ipsilesional gain of 0.07 and a contralesional gain of 0.16; the subjective visual vertical was 7.8°. The gains measured all impulses delivered during HITD testing were 0.14 ± 0.07 ipsilesionally and 0.74 ± 0.04 contralesionally. In the normal subject (Figure 5A) the eyes rotate in the opposite direction of the head (with the same velocity), gaze (Figure 5C) remains basically stable and the subject is indeed able to correctly recognize the optotype.

In the patient response (Figure 5B), the eyes move together with the head and saccades are triggered to regain fixation. The first is a covert saccade occurring during the presentation of the optotype with a latency of about 100 ms from the onset of the head movement, while the second is an overt saccade allowing the patient to regain fixation. Compared with the healthy subject, the gaze of the patient (Figure 5D) is not stable during the optotype presentation (Figure 5), and his answer was indeed not correct. Gaze returns to the target, at 0°, only after the end of head rotation.

A further context of application for the proposed analysis tool is the study of the patient follow up during recovery and rehabilitation, in which the performed measurements allow to assess the progress of the VOR response and the contribution of the saccadic component, while studying their correlation with the improvement in the rate of correctly identified optotypes.

**Figure 6:**
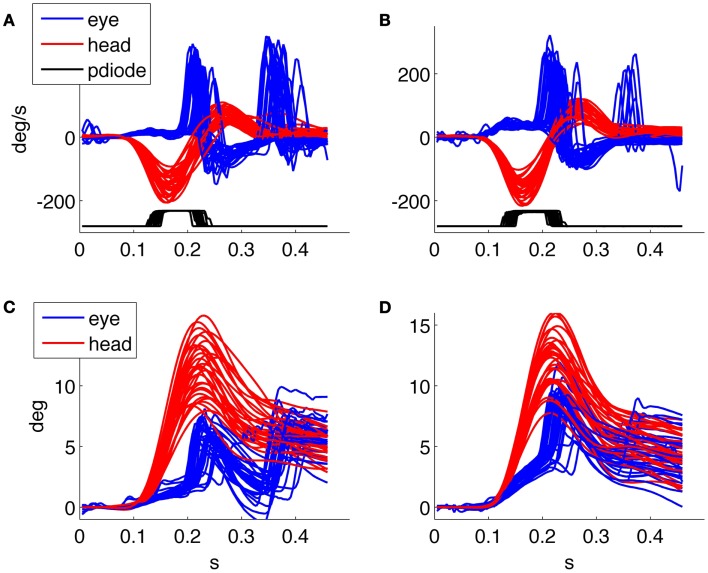
**Head impulse testing device test 3 days (on the left) and 1 month (on the right) after the acute phase of a vestibular neuritis**. **(A,B)** velocity plot, **(C,D)** position plot. The patient improved the percentage of correct answers from 3 to 39%.

For example, Figure 6 shows the responses of the same patient with right vestibular neuritis that we already showed in Figure 5, to compare the recordings 3 and 30 days after the disease onset. After 30 days her ipsilesional gain value at rotatory testing was 0.1, her contralesional gain value 0.18, and the subjective visual vertical was 5.6°. With HITD testing her gain values were 0.30 ± 0.03 ipsilesionally and 0.82 ± 0.03 contralesionally. In the follow up recording the patient had an improvement of the slow phase gain and he reduced the number and the amplitude of overt saccades, intensifying the use of covert saccades. The patient improved the percentage of correct answers with ipsilesional head impulses from 3% during the first examination to 39% during the last. The analysis of individual trials for verifying gaze behavior with respect to optotype timing, together with the knowledge of the subject’s answer, will allow further insight on these recovery processes.

## Discussion

Functional testing of the VOR has recently been proposed as a simple and reliable means for testing the peripheral vestibular system without computing the gain of the VOR and thus without the need for eye movement recording. In spite of the attention that these techniques have raised, many of the relevant parameters regulating the testing procedure differ significantly among the approaches proposed in the literature and their role and optimal values have not been understood yet. The lack of studies systematically facing these issues has thus far been due, we believe, to the lack of a tool allowing to reliably collect all the necessary, synchronized data. In fact the search coil technique, gold standard for research in the field, is not applicable within a functional testing context since eye coils and the topical anesthetic that is typically used to reduce discomfort, seriously impair clear vision. On the other hand, the recently proposed video systems able to record eye movements during HIT (20) have not been used in closed loop (i.e., exploiting the recorded head velocity for triggering stimuli) for functional testing paradigms thus far, and they do not allow the recording of stimulus timing information. Our experience with the 60 Hz monitor reported in the results section is an example of how critical it may be to verify the timing of the visual stimuli. For these reasons, current tools do not allow to study the role of covert saccades toward functional recovery either, as information on when the saccade occurs with respect to the appearance of the optotype on screen is not available. Thus, we have developed a new research tool for the thorough analysis of functional testing of the VOR, which for the first time allows to record synchronized data on head rotations, eye movements, and the appearance of the visual stimuli on screen. The tool may be used to implement any of the functional testing techniques proposed in the literature and described in the introduction. Here, we have presented some preliminary results obtained using such new system with the HITD paradigm on a few patients and healthy subjects with the purpose of illustrating the innovative potential of the proposed research tool.

## Conflict of Interest Statement

The authors declare that the research was conducted in the absence of any commercial or financial relationships that could be construed as a potential conflict of interest.
